# Infants’ expectations about gestures and actions in third-party interactions

**DOI:** 10.3389/fpsyg.2014.00321

**Published:** 2014-04-22

**Authors:** Gudmundur B. Thorgrimsson, Christine Fawcett, Ulf Liszkowski

**Affiliations:** ^1^Communication Before Language Research Group, Max Planck Institute for PsycholinguisticsNijmegen, Netherlands; ^2^Uppsala Child and Baby Lab, Department of Psychology, Uppsala UniversityUppsala, Sweden; ^3^Department of Developmental Psychology, University of HamburgHamburg, Germany

**Keywords:** third-party interactions, action understanding, eye tracking, infants, gestures, object-directed actions

## Abstract

We investigated 14-month-old infants’ expectations toward a third party addressee of communicative gestures and an instrumental action. Infants’ eye movements were tracked as they observed a person (the Gesturer) point, direct a palm-up request gesture, or reach toward an object, and another person (the Addressee) respond by grasping it. Infants’ looking patterns indicate that when the Gesturer pointed or used the palm-up request, infants anticipated that the Addressee would give the object to the Gesturer, suggesting that they ascribed a motive of request to the gestures. In contrast, when the Gesturer reached for the object, and in a control condition where no action took place, the infants did not anticipate the Addressee’s response. The results demonstrate that infants’ recognition of communicative gestures extends to others’ interactions, and that infants can anticipate how third-party addressees will respond to others’ gestures.

## INTRODUCTION

Infants are highly attuned to others’ actions. In their first year, they interpret both instrumental actions, such as reaching, and communicative actions, such as pointing, as goal-directed behavior (Woodward and Guajardo, 2002). For example, they expect others to consistently reach for the same object (Woodward, 1998; Cannon et al., 2012) and to reach in an efficient manner given the environment (Brandone and Wellman, 2009). They follow pointing gestures to objects (Deák et al., 2000) and can infer the social motive of a point based on social contextual cues (Behne et al., 2005; Liebal et al., 2009). Understanding actions in terms of goals or motives enables infants to learn about actions and objects through observation and interaction, and to engage in relatively complex non-verbal communicative interactions. However, although the focus of the majority of research on communication and action understanding in infancy is on dyadic settings – where the infant observes or engages with another person in a one-on-one exchange – these represent only a part of infants’ early communicative experience. During their first year infants also routinely observe and overhear communicative interactions between other people that provide infants with another source of information about the social world. In traditional cultures where preverbal infants are rarely directly addressed by caregivers (e.g., the Tzeltal Mayans: Brown, 1998; See also Lieven, 1994), such observational experiences could play a particularly important role in infants’ social and communicative development.

Recent experimental research indeed shows that infants monitor and learn from actions that are not directed at or addressed to them, but that they observe and overhear being addressed to a third party. The bulk of this research focuses on infants’ ability to learn words or actions used in third-party interactions. For example, 18-month-olds can learn object labels through overhearing the labels being used in others’ interactions (Floor and Akhtar, 2006), even when the objects are labeled only indirectly (Gampe et al., 2012). Studies on imitative learning similarly show that infants at this age will imitate a novel action demonstrated to a third party (Herold and Akhtar, 2008; Matheson et al., 2013) and even attempt to imitate the social nature of an action demonstration (Fawcett and Liszkowski, 2012).

It is less clear, however, how much infants understand about the structure and outcome of third-party communicative interactions. Such understanding would entail not only an ability to imitate or learn from actions addressed to third parties, but to anticipate how the addressees respond. A few recent studies suggest that infants have expectations toward addressees of speech in third-party interactions. Specifically, 12- and 24-month-olds are quicker to shift their gaze from a person to a third-party addressee when the person utters speech, than when the person emits natural non-speech sounds, suggesting a stronger expectation of a response to the former (Thorgrimsson et al., 2011). A looking time study further revealed that 12-month-old infants expect addressees to respond to speech in accordance with the speaker’s previous object-directed actions, suggesting that infants recognize that speech can transfer information about an object (Martin et al., 2012). Infants also seem to have some understanding of the use of gestures in third party communication. When presented with a scene where one person indicates the location of a hidden toy to a third-party addressee by pointing at it in a communicative fashion, infants were able to locate and retrieve the toy themselves (Gräfenhain et al., 2009). Together, these studies suggest that infants have expectations regarding the addressee of speech in third-party interactions and that they can pick up on information conveyed through gestures. It remains unknown, however, whether infants can also anticipate the actions of the addressee of gestures in third-party interactions.

The current study used eye tracking to examine infants’ expectations toward third-party addressees of gestures and actions. 14-month-olds watched a third-party interaction where one person (the *Gesturer*) directed a gesture (a point or a palm-up “request” gesture) or an instrumental action (a reach) to an object between them. The other person (the *Addressee*) then responded by grasping the object and dispensing it through one of two tubes that led to each person, thereby either giving the object to the Gesturer, or taking it for himself. As the Addressee’s hand and the tube entrances were concealed by an occluder, the infants could not see into which tube he placed the ball, and had to look at the tube exit to see the outcome. Importantly, the Gesturer did not verbally communicate any motive, and as both actors were visible only from the neck down, no information could be gleaned from their facial expressions or their gaze direction.

We expected that the nature of each of the three demonstrated actions would lead infants to anticipate different outcomes. A point is a deictic gesture that gets its meaning from the social context within which it is used. In the context of the study, the point can be construed either as communicating a request for the object, or an offer for the Addressee to take it. As infants point for various social motives, including requesting, sharing interest, and informing (e.g., Liszkowski et al., 2006), and their interpretation of others’ points depends on the social context in which they take place (Behne et al., 2005, 2012; Liebal et al., 2009), they may be ready to interpret the pointing gesture in the current third-party context both as communicating a request for the object, and an offer for the Addressee to take it, leading them to anticipate both outcomes. The request gesture, on the other hand, is a conventional gesture communicating a request to hand over an object, which caregivers use from early on (Bruner, 1977) in give-and-take exchanges, and which infants recognize and respond to (Hay and Murray, 1982; Thorgrimsson and Liszkowski, 2012). If infants’ recognition of the request gesture extends to third-party interactions, they should only anticipate the give outcome. Finally, infants start making reaching attempts at around 4 months of age (von Hofsten and Lindhagen, 1979), and there is a lot of evidence that infants expect others’ reaching attempts to indicate goal-directed behavior, both from action-processing studies (e.g., Woodward, 1998; Woodward and Guajardo, 2002; Cannon et al., 2012) and interaction-based studies (e.g., Warneken and Tomasello, 2007). Although infants will likely understand the reaching action in the current study as an instrumental attempt to grasp the object, a reach is typically not used to communicate a request, and thus it is unclear if infants see it as having relevance to the Addressee. However, it is also possible that infants expect people to help others achieve instrumental goals and thus expect the Addressee to give the object. These three actions were compared to a control condition where the Addressee did not gesture but remained silent and immobile for an equal duration of time.

## MATERIALS AND METHODS

### PARTICIPANTS

Eighty 14-month-old infants (41 boys, 39 girls; mean age = 14:17, range = 13:24–14:29) participated in the experiment. An additional 19 infants participated but were not included in the final sample: twelve due to not finishing the experiment as a result of fussiness, five due to watching less than 25% of the demonstration phase, and two due to technical issues. Infants were recruited from a database of families who expressed interest in participating in research. Infants were primarily white and from middle-class backgrounds, living in a medium-sized European city. Parents received a small gift for participating.

### APPARATUS

Infants’ eye movements were measured with a Tobii T120 remote eye tracker, using a sampling rate of 60 Hz. The eye tracker has an accuracy of 0.5°, precision of 1°, and allows head movements of up to 44 cm horizontally, 22 cm vertically, and 30 cm in depth. The eye tracker is integrated with a 17 inch TFT display with a native resolution of 1280 × 1024 pixels.

### STIMULI AND DESIGN

The study had a between-subject design with four groups of 20 infants each. In the experimental groups the Gesturer pointed, reached, or directed a request gesture toward the object, but in the no-gesture control group no action was performed. Infants were presented with a total of 13 videos (29.50 × 14.32 visual degrees excluding black bars at the top and bottom of the screen), which collectively lasted approximately 5 min. The videos showed two people (Gesturer on left and Addressee on the right) in profile sitting at a table, with a small black shelf between them. A transparent tube ran from each side of the shelf to a bowl in front of each person.

**FIGURE 1:**
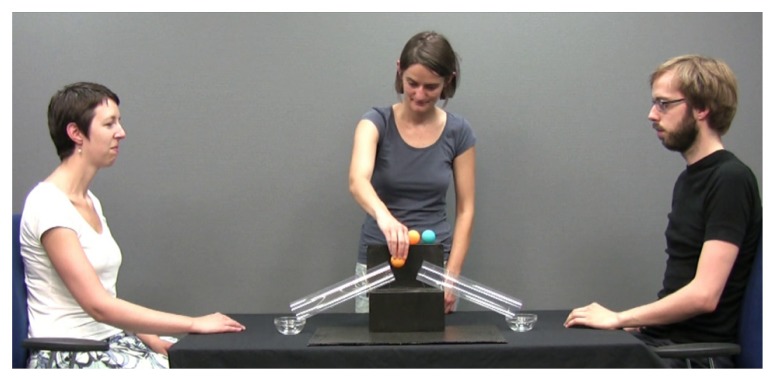
**A frame from the demonstration phase showing the Demonstrator distributing balls**.

As the tubes would be occluded from view during the test trials, infants in all groups first viewed a *demonstration *video without an occluder where a third person (the Demonstrator) stood behind the shelf and distributed balls through the tubes demonstrating how the tubes functioned (see **Figure 1**). In the demonstration, the Demonstrator picked up a ball from the top of the shelf, looked at the Addressee or Gesturer (whichever would receive the ball that time) with a brief smile, and placed the ball into the tube leading to his or her bowl. The Demonstrator distributed four balls in this way (two blue and two yellow), alternating between the Addressee and the Gesturer. Using video editing software, the ball’s movement through the tube was slowed down such that it took 1 s to travel through the tube, and was accompanied by a previously recorded sound of a ball rolling through a long paper tube. To give the impression that the two people were interested in the balls, they responded by picking up the ball, looking at the Demonstrator and vocalizing happily. The demonstration phase had a duration of 69 s and ended with the Demonstrator placing an occluder in front of the shelf.

**FIGURE 2:**
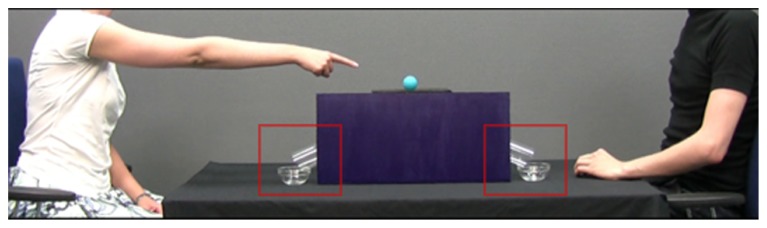
**A frame from the test trial in the point group, showing the Gesturer pointing to the ball**. The two red squares delineate the areas of interest.

Next, each of the 12 *test trial* videos revealed the Gesturer and the Addressee seated as before and a single ball on top of the shelf. The upper parts of the tubes were concealed by the occluder, such that only the tube exits could be seen protruding from behind it. To direct infants’ attention away from the actors’ faces and to their actions, and to prevent infants from detecting their gaze direction, the actors were visible only from the neck down. In the experimental groups, the Gesturer directed an action (point, request, or reach) toward the ball and sustained it for 2 s before retracting her arm (see **Figure 2**). As the extension of the Gesturer’s arm was identical in all three experimental groups, the only difference between groups was the Gesturer’s hand shape. In the no-gesture control group, the Gesturer did not act toward the object, but sat immobile for the same duration of time. In all four groups, the Addressee then responded by reaching for and picking up the ball, and placing it into one of the tubes. As the Addressee’s hand and the tube entrances were concealed by the occluder, the infants could not see into which tube he placed the ball, and would have to look at the tube exit or the bowl to see the outcome. As in the demonstration phase, the balls’ movement through the tube was artificially slowed down. Four seconds elapsed from the time the Addressee’s hand disappeared behind the occluder and until the ball emerged from the tube. To facilitate anticipation, the last 2 s of the ball’s movement were accompanied by the same sliding sound as in the demonstration phase. For each group, in one half of the test trials the ball emerged on the side of the Gesturer (*Give* trials) and in the other half on the side of the Addressee (*Take* trials). The twelve test trials were presented as blocks of six Give trials and six Take trials, with block order counterbalanced across infants. The first block always featured blue balls and the second yellow balls, so that for each infant, Give and Take trials were also distinguishable by the color of the balls.

### PROCEDURE

Infants were seated in a safety car seat that was placed in their parents’ lap so that the infants’ eyes were approximately 60 cm from the monitor. Before the experiment the infants’ gaze was calibrated using a 9-point calibration during which the experimenter monitored the infants’ attention on the screen and showed a short animated video of talking puppets in place of the calibration animation to recapture attention when needed. Between trial blocks, a short (4s) animated video (a small shaking cartoon bird accompanied by sound) was played to sustain the infants’ attention. Audio was transmitted through a single desktop speaker connected to the computer and hidden from view behind the monitor.

### DATA REDUCTION

Rectangular areas of interest (AOI’s) were created covering the exit of the tube and the bowl on each actor’s side (3.30 × 2.83 visual degrees each; see **Figure 2**). Infants’ raw gaze data points registered within these AOI’s at a rate of 60 per second were used to calculate the dependent measures. To account for possible errors in gaze estimation, the AOI’s covered an area approximately 30 pixels (0.8 visual degree) wider and higher than the tubes and bowls (e.g., Gredebäck and Melinder, 2010). The time window selected for analysis – the anticipatory phase – was calculated by finding the frame when the Addressee touched the ball and the frame when the ball emerged from a tube (5.5 s). As the time it takes to initiate a saccade is around 200 ms (Becker, 1972; Canfield et al., 1997), the anticipatory phase was shifted forward by 200 ms from the onset time of these two frames. Although infants were not expected to anticipate the emergence of the ball from the tube until the Addressee’s hand had disappeared behind the occluder, it is possible that they expect the Addressee to manually transfer the ball into either bowl, and thus the anticipatory phase started as soon as the Addressee grasped it.

Two dependent measures were extracted from the gaze data. The proportional looking time measure was calculated by dividing the looking time to the Gesturer’s AOI by the total looking time to both AOI’s during the anticipatory phase. Looking time was calculated by summing the gaze data points that fell within the AOI’s. The first look measure is a simple binomial measure that specifies which AOI the infant looked at first. It was calculated by subtracting the time at the onset of the anticipatory phase from the time at which the first gaze data point was first registered within each AOI (the latency) and selecting the AOI with the shorter interval. If infants anticipate that the ball will emerge on the Gesturer’s side, then their proportional looking time and their first looks to the Gesturer’s AOI should be greater than chance (50%).

## RESULTS

As the measures tended to be skewed, and tests for normality (Kolmogorov–Smirnov) revealed that the measure of first look deviated from normality for the Give outcome in the point group [*D*(20) = 0.232, *p* = 0.006] and marginally so for the Take outcome in the Reach group, [*D*(20) = 0.221, *p* = 0.011], non-parametric tests were used. The main analyses are one-sample Wilcoxon signed-rank tests, measured against chance levels (a median of 0.5). The Bonferroni correction was used to correct for multiple comparisons, yielding a two-tailed significance threshold of 0.0063.

### REQUEST

**FIGURE 3:**
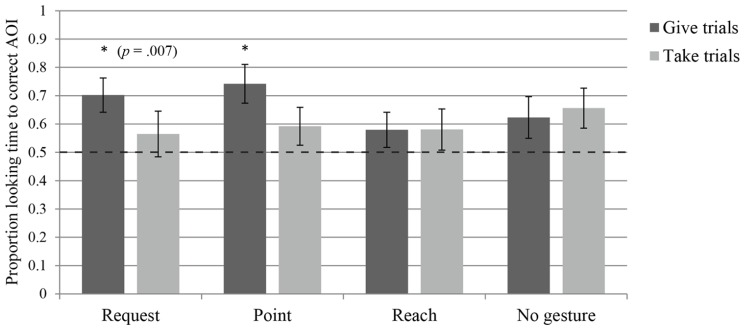
**Average proportion of looking time to the target bowl AOI during the anticipatory phase for each of the four groups, separated into give trials – where the Addressee gave the object to the Gesturer – and take trials – where the Addressee took the object for himself.** Dashed line indicates chance level and asterisks indicate a significant difference from chance (*p* < 0.0063, unless otherwise specified).

**FIGURE 4:**
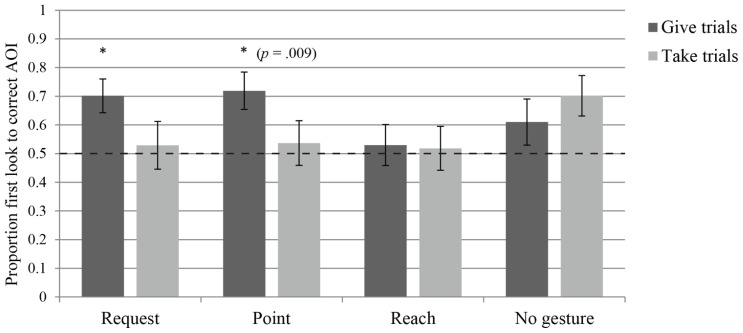
**Average proportion of first looks to the target bowl AOI during the anticipatory phase of each of the four groups, separated into give trials – where the Addressee gave the object to the Gesturer – and take trials – where the Addressee took the object for himself.** Dashed line indicates chance level and asterisks indicate a significant difference from chance (*p* < 0.0063, unless otherwise specified).

Infants in the request group looked at at least one of the AOI’s during the anticipatory phase in 73.75% of the trials (SD = 19%). The measure of proportional looking time to the Gesturer’s bowl in the Give and Take trials revealed that the infants tended to look longer at the bowl into which the ball emerged (the *target bowl*) in the Give trials (*Median* = 0.80, *W* = 162.000, *p* = 0.007), but not in the Take trials (*Median* = 0.51, *W* = 114.500, *p* = 0.432), indicating that they only anticipated the give outcome (see **Figure 3**). The first look measure yielded the same results as the looking time measure: the infants looked first at the target bowl in the Give trials (*Median* = 0.80, *W* = 124.000, *p* = 0.003), but not in the Take trials (*Median* = 0.50, *W* = 74.000, *p *= 0.754; see **Figure 4**). Thus, although one measure is marginally significant, both indicate that infants in the request group anticipated the give outcome, whereas neither measure indicates that they anticipated the take outcome. To assess learning across the first block of trials, regression analyses were performed on both outcomes for both measures. Proportional looking time was not found to change significantly across trials for the Give outcome [β = 0.019, *t*(47) = 0.558, *p* = 0.58], or for the Take outcome [β = 0.101, *t*(40) = 2.416, *p* = 0.02]. Similarly, the first look measure did not indicate learning across trials for the Give outcome [β = 0.148, χ2(1) = 0.474, *p* = 0.491], or the Take outcome [β = 0.395, χ2(1) = 3.719, *p* = 0.054]. Thus the infants do not seem to be learning to anticipate the outcomes over the course of the trials, but rather have an *a priori* expectation regarding the request gesture that leads them to anticipate the give outcome.

### POINT

Infants in the point group looked at at least one of the AOI’s during the anticipatory phase in 64.58% of the trials (SD = 17.70%). The measure of proportional looking time to the Gesturer’s bowl revealed that they looked longer at the target bowl in the Give trials (*Median* = 0.79, *W* = 158.000, *p* = 0.002), but not in the Take trials (*Median* = 0.55, *W* = 117.500, *p* = 0.162), indicating that they only anticipated the give outcome (see **Figure 3**). The first look measure similarly revealed that infants had a tendency to make more target first looks only in the Give trials (*Median* = 0.75, *W* = 105.000, *p* = 0.009), but not in the Take trials (*Median* = 0.50, *W* = 86.000, *p* = 0.650; see **Figure 4**). Thus, even though one measure was only marginally significant, both indicate that infants in the point group anticipated the give outcome, but not the take outcome. To assess learning across the first block of trials, regression analyses were performed on both outcomes for both measures. Proportional looking time was not found to change significantly across trials for the Give outcome [β = 0.037, *t*(32) = 0.93, *p* = 0.359], or for the Take outcome [β = 0.042, *t*(43) = 1.014, *p* = 0.316]. Similarly, the first look measure did not indicate learning across trials for the Give outcome [β = 0.208, χ2(1) = 0.505, *p* = 0.477], or the Take outcome [β = 0.076, χ2(1) = 0.146, *p* = 0.702]. Again, this indicates that infants did not learn to anticipate the outcomes during the experiment.

### REACH

Infants in the reach group looked at at least one of the AOI’s during the anticipatory phase in 63.30% of the trials (SD = 19.48%). The measure of proportional looking time to the Gesturer’s bowl revealed that infants did not looked longer at the target bowl in the Give trials (*Median* = 0.62, *W* = 95.000, *p* = 0.162), or in the Take trials (*Median* = 0.50, *W* = 93.000, *p* = 0.194; see **Figure 3**). The first look measure similarly revealed that infants did not look first at target bowl in the Give trials (*Median* = 0.60, *W* = 77.500, *p* = 0.621), or in the Take trials (*Median* = 0.50, *W* = 43.500, *p* = 0.715; see **Figure 4**). Thus, both measures indicate that infants in the reach group failed to anticipate either outcome. To assess learning across the first block of trials, regression analyses were performed on both outcomes for both measures. Proportional looking time was not found to change significantly across trials for the Give outcome [β = 0.082, *t*(30) = 1.643, *p* = 0.111], or for the Take outcome [β = 0.019, *t*(36) = 0.54, *p* = 0.593]. Similarly, the first look measure did not indicate learning across trials for the Give outcome [β = 0.29, χ2(1) = 1.407, *p* = 0.236], or the Take outcome [β = -0.162, χ2(1) = 0.421, *p* = 0.516]. As for the previous gestures, no learning over trials was apparent for infants.

### NO GESTURE

Infants in the no gesture group looked at at least one of the AOI’s during the anticipatory phase in 52.94% of the trials (SD = 22.40%). The measure of proportional looking time to the Gesturer’s bowl revealed that infants did not look significantly longer at the target bowl in the Give trials (*Median* = 0.59, *W* = 107.000, *p* = 0.144), or in Take trials (*Median* = 0.71, *W* = 106.000, *p* = 0.049), indicating that they did not anticipate either outcome (see **Figure 3**). The first look measure revealed that infants did not look first at the target bowl at higher than chance levels in the Give trials (*Median* = 0.600, *W* = 92.500, *p* = 0.192), or in the Take trials (*Median* = 0.73, *W* = 68.000, *p* = 0.019; see **Figure 4**). Thus, both measures indicate that infants in the no gesture group failed to anticipate either outcome. To assess learning across the first block of trials, regression analyses were performed on both outcomes for both measures. Proportional looking time was not found to change significantly across trials for the Give outcome [β = -0.041, *t*(29) = -1.086, *p* = 0.287], or for the Take outcome [β = 0.029, *t*(37) = 0.798, *p* = 0.430]. Similarly, the first look measure did not indicate learning across trials for the Give outcome [β = -0.272, χ2(1) = 0.941, *p* = 0.332], or the Take outcome [β = 0.086, χ2(1) = 0.144, *p* = 0.704]. With no gesture present, infants also did not show a change in response over trials to indicate that they were learning from the observed outcomes.

## DISCUSSION

We found that 14-month-old infants expected a third party addressee to produce a specific action in response to another person’s communicative gestures. As neither the motive behind the person’s gesture, nor the intention of the addressee could be inferred from their gaze direction or facial expressions, the infants’ expectations toward the addressee were based only on the hand shape of the person’s gesture. When the Gesturer pointed or directed a palm-up request gesture toward an object located between them, the infants expected the Addressee to give the object to her. Infants’ expectations are reflected in their ability to anticipate that the Addressee will respond to the gestures by transferring the object to the Gesturer through a tube, and their inability to anticipate the opposite response of transferring the object to himself. Importantly, infants in a control group where no action was produced did not show evidence of anticipating the Addressee’s response, ruling out the possibility that infants can anticipate the Addressee’s actions without recognition of the gesture. In the reach group, where the Gesturer reached toward the object, infants also failed to anticipate the actions of the Addressee, possibly because they did not understand the reaching as a communicative action.

The current study complements previous findings that infants’ understanding of communicative gestures is not restricted to dyadic settings, but extends to observed third-party interactions (Gräfenhain et al., 2009). The current findings further reveal that infants monitor and show understanding of non-verbal third-party interactions not only when the interaction has relevance to their own desired goal (as in Gräfenhain, 2009), but also when they are passive observers. Moreover, mirroring findings on infants’ expectations about speech directed to third parties (Thorgrimsson et al., 2011; Martin et al., 2012), the study indicates that infants have specific expectations about the actions of third-party addressees of communicative gestures.

The findings indicate that infants’ expectations about the interactions were in place before they first observed the outcome. Given that infants’ anticipatory looking did not show a consistent change across the first block of trials, their expectations do not seem to have developed through repeated viewings of the outcome (in which case they would be based on infants’ readiness to learn to associate certain actions with a specific outcome). It should also be noted, however, that although infants anticipated the give outcome in the give trials, their performance was at chance for the take trials, suggesting that their expectations are not robust enough to hold up to repeated evidence that the Addressee will take the object for himself.

From these findings, we cannot yet determine how infants develop expectations regarding these gestures. Their experience observing third-party interactions and their own dyadic interactions may both be contributing factors. The novelty of the interaction presented in the current study makes it unlikely, however, that their expectations are the product only of associative learning from observing the sequence of events in previous third-party interactions. The interaction we presented featured a novel and indirect means to transfer the object and neither participant presented any ostensive cues, yet infants anticipated the addressee’s response. This suggests that infants’ understanding of the function of these gestures is abstract enough for their expectations to be generalized across different contexts. This is in line with results from a recent interaction-based study showing that infants respond appropriately to the point and the palm-up request even in the absence of accompanying social-contextual cues (Thorgrimsson and Liszkowski, 2012).

It is interesting to speculate how infants’ third-party expectations may relate to their own frequent experience with these gestures in communicative interactions. Despite the fact that infants’ own pointing, and their interpretation of others’ points shows that they are aware of the gesture’s potential to communicate a variety of social motives, the infants were prepared to construe the point in the current third-party context as an object request, but not as an offer for the Gesturer to take the object. However, although we know of no relevant empirical data, it is highly likely that infants point more frequently to request objects than to offer them, which may shape their interpretation of the function of points in others’ interactions. Regarding the palm-up request, infants’ anticipation in the request group suggests that infants expect its hand shape it to indicate an object request, even when they encounter the gesture outside a dyadic interactive context. As infants are very rarely observed to use this gesture to request (Hay and Murray, 1982; Bakeman and Adamson, 1986; Puccini et al., 2010), their expectations more likely stem from their experience as addressees in the give-and-take routine. Regarding the reach, infants’ failure to anticipate the outcome in the reach group suggests that they did not expect the action to communicate a request to the Addressee, or to provoke the Addressee to help. However, the nature of the reach leave the results open to an alternative explanation. By coming very close to reaching the object but then retracting her arm, the Gesturer may have given the impression that she could have obtained the object but chose to leave it in its place. It is important to note, however, that this procedure was identical in the other groups, yet the infants were able to recognize the gestures as communicating a request for the object.

Together with recent research on infants’ expectations about speech directed to a third party (Thorgrimsson et al., 2011; Martin et al., 2012), the current findings indicate that infants monitor third-party interactions and have specific expectations regarding addressees already at 14 months of age and possibly earlier. Attending not only to people’s actions, but to the reactions they provoke from addressees, is likely to expand infants’ opportunities for social-observational learning. For example, attending to the contribution of both interactants in social interactions should help infants learn from others’ joint cooperative activities. The findings also raise the question of whether the arguably more demanding skill of learning novel object labels and novel arbitrary actions from third-party interactions may also emerge by this age. The fact that infants in the current study predicted the addressee’s response to the gestures suggests that they may have taken his perspective into account when interpreting the actions. One theory of social-cognitive development posits that the ability to understand others’ interactions from the perspective of the interactants is precisely what enables infants to learn from third-party interactions (Moore, 2007). Although there is some indication that learning novel actions is still an emerging skill at 18 months (Floor and Akhtar, 2006; Herold and Akhtar, 2008), a recent study shows word learning at 18 months even under difficult conditions (Gampe et al., 2012). Future research should examine younger infants’ ability to learn words and actions from others’ interactions.

The current findings demonstrate that infants have expectations about how third-party addressees respond to common gestures in an interaction. Further, they show that infants’ recognition and use of gestural information is not limited to interactions that have direct relevance to infants’ own goals. The ability to understand and predict others’ social interactions is valuable as it offers infants the opportunity to learn through observing and overhearing the many interactions that surround them every day.

## Conflict of Interest Statement

The authors declare that the research was conducted in the absence of any commercial or financial relationships that could be construed as a potential conflict of interest.
